# Report on Practical Medicine

**Published:** 1868-04

**Authors:** S. P. Breed


### CHICAGO MEDICAL EXAMINER.

N. S. DAVIS, M.D., Editor.

VOL. IX. APRIL, 1868. NO. 4.

### Original Contributions.

### ARTICLE XI.

### REPORT ON PRACTICAL MEDICINE.

By S. P. BREED, M.D.

Read to the Military Tract Medical Society.

*Gentlemen of the Military Tract Med. Society:*—In behalf of the Committee on Practical Medicine, allow me to remark that my report consists of two papers, one, a brief, hurried, and, therefore, very imperfect retrospect of the past year, and some observations relative to the diseases peculiar to this climate, as they appear to one who has recently come here. The other paper consists of an essay read before the State Medical Society, at Decatur, in 1866, with the request that it should not be published; together with a polypus of the uterus, and its history.

REPORT PROPER.

The current year has been noted in Northern Illinois for extraordinary good health. No marked epidemic of any kind as far as my knowledge has extended, has entered to dissurb the universal tranquility throughout the agricultural districts of this part of the State.

Early in the season, much apprehension was felt, everywhere, amongst all classes, lest the cholera, which seemed to be steadily approaching us, should invade the quietude of our peaceful and happy homes in this section. With the view of mitigating the evil as much as possible, the Town Council bestirred themselves; the sanitary rules and regulations were reviewed; health officers employed; nuisances abated, as much as possible, so as to be in readiness for the fearful scourge. Nevertheless, the hot season came and went; men hurried, women worried, but the dread epidemic kept at a respectful distance. Other diseases seemed to retire at its approach, as though they would give it an open field and undisputed possession of the ground; and *all*, by a sort of common consent, seemed to look with fearful dread and apprehension, expectant of the unwelcome visitant. But, fortunately, it came not.

There have been, by far, fewer diseases common to the sultry days of summer and the "sere and yellow leaf" of autumn, than for many years past. Cholera-infantum, which usually carries off its victims in such frightful numbers in our cities, towns, and even in the country districts, has been almost unknown amongst us the past season.

If I were asked to state the nature of the diseases prevalent here during the past summer, I would say that they were almost entirely of a nervo-rheumatic, neuralgic, or a hysterical character, or non-inflammatory. Indeed, inflammatory affections have been of exceedingly rare occurrence. The few fevers that have presented themselves to my observation, have generally been of a mild intermittent type, or, at least, have not been disposed to be continued. It is true, however, that, recently, several more severe and obstinate cases of a *low* and *continued type* have fallen under my observation, and they have seemed to me to approach that form of fever called cerebro-spinal meningitis, or spotted fever; but, they have, I am happy to say, been very few.

The season has been one of excessive *drouth*, especially during the latter part of the summer and fall. No rains have fallen, of any consequence, since the harvest. The temperature has been uniformly mild and pleasant, and the frosts of autumn have delayed their coming much later than usual. Fruits of all kinds were very abundant, of which our people partook without stint or moderation. Crops of all kinds came in good; and every department of useful industry and art flourished, and all classes (except the doctors) reveled in a universal cornucopia.

Coming, as I did, from a latitude some 3° south of this, it may not be uninteresting to medical men here to state what my impressions are, in regard to the variation in the character of the diseases as they prevail in the two sections. One of the first things observed by me, after my arrival in this climate, was a remarkable tendency, among the people generally, to a diarrhœa. Purgative medicines acted much more easily and freely than I had been in the habit of seeing them do. Doses no larger than I had been in the habit of using constantly, I found, often, to act with extreme energy, producing drastic catharsis; while diarrhœas proved peculiarly obstinate, and sometimes quite intractable, the acute stage inevitably passing gradually into the chronic. So noticeable was this feature of difference in the character of the same diseases in the two localities, that those symptoms which I should have considered trivial in my former place of residence, often proved tedious, troublesome, and sometimes bid defiance to all methods of treatment, carrying the patient along for weeks, and months even, in a state of painful anxiety and impaired activity. This peculiarity, I doubt not, affords an explanation for the fact that some of our physicians have, they say, pretty much ignored the use of cathartics in their practice. Now while I see and acknowledge this tendency to diarrhœa, and allow that this fact should be remembered in all of our prescriptions, I, nevertheless, with all due deference to their opinions, still think they go to an opposite extreme, in rejecting purgatives from their list of remedies. "*In medio tutissimus ibis*," is a maxim as true in the practice of medicine as in the nicest deductions of transcendental philosophy.

In all of those cases where this tendency to diarrhœa obtains *decidedly*, these physicians may succeed very well; but I have good reason to know that it now and then happens that they meet with cases of habitual constipation, in which their set of remedies not only do no good, but, in all probability, do much harm.

For instance, I have known a few cases (treated ineffectually by the advocates of the non-cathartic plan) of pregnant women passing on through the latter part of their period of utero-gestation, with such a distressing state of obstinate constipation that, when the critical period of accouchement came, their whole systems were borne down by the depressing effects of the absorption into the circulation of much of those deleterious and noxious matters which instead of passing off through the natural channel, the alimentary canal, were diverted *therefrom*, and, by the law of compensation, were attempted to be removed through the absorbents and exhalants. Before I proceed further on this point, I wish to remark that, very soon after I came here, I observed, also, that there were very many more cases of spinal disease than I had been accustomed to witness. I was at a loss to account for this phenomenon. Whether it arose from the difference in the occupation and character of the people, or was due solely to local or climatic causes, I could not tell; but of the fact, I have no doubt. By calling the attention of medical men to the matter, I hope to obtain such assistance as will lead to a plausible explanation. Some of these cases, I thought, merited the name of spinal irritation, while others were attended with symptoms so different and unique, that I was sometimes at a loss to determine what was the appropriate appellation for them. Then, we had spinal meningitis, both acute and chronic. Dr. Wood thinks chronic meningitis a very rare disease. I am inclined to believe that, in this climate, it is not so very rare. Then, we have those symptoms which induce the belief that the case is one of myelitis and its results. Sometimes, the inflammations were undoubtedly of a gouty or rheumatic character. Now, amid all these different varieties of spinal complaints, we had cases of paralysis of the limbs, atrophy, and degeneration of the muscles of the extremities, so that the halt, tottering, and deformed met me at every corner.

This general tendency to spinal complaints, I think, may probably account for another circumstance which I soon observed, and that was, that strychnia, nux vomica, ignatia, and brucia, together with valerian and such nerve stimulants and sedatives, were in use to a much larger degree than I had been accustomed to see them. They were often used, indeed, in diseases where there did not appear to be any demand for them. They, or some of these active remedies, were almost sure to enter into every cathartic pill, and, frequently, into mixtures for diarrhæa, dyspepsia, neuralgia, hysteria, dysmenorrhæa, and amenorrhœa, etc.

In passing, I mention another item of some note. In a practice of some eighteen years where I formerly resided, I never happened to meet with a case of tapeworm; but, during the two years of my stay in this locality, though my means of observation have been much more limited, I have seen nearly half a dozen cases; while intestinal worms, generally, are more frequently met with. The people in the former place live much on pork, while the inhabitants of this neighborhood pretty generally use beef. Two of the cases, I found occurred in persons who seldom, if ever, ate pork, but, as they said, were "great on rare beefsteak." What connection this circumstance may have with the origin of the tænia I do not know, but I mention it, that it may go for what it is worth. It may mitigate the prejudice against pork eating, perhaps, and tend to throw some suspicion on the healthiness of raw beef. It may not be amiss, also, to mention the circumstance related by Hasselquist, that the tapeworm is very common in Egypt, among the Jews, who, it is well-known, are religiously restrained from eating pork.

I continually meet persons *here*, afflicted with nasal catarrh and œzena. The per cent of such cases must be much greater here than where I formerly lived.

It is here that ignorant, itinerant charlatans with their electro-galvanic machines, like organ-grinders, who pretend to grind diseases of all kinds out of their patients, meet with extraordinary success, and electrical baths are immensely popular. From a somewhat careful survey of all these phenomena, I am inclined to believe that, in order to understand this seemingly complex and difficult subject, we must look to the cerebro-spinal and ganglionic system of nerves. I apprehend that the remote cause of many of these cases of obstinate constipation, in females especially, may be found in a depressed state of the nerve centres, particularly those sympathetic ganglia supposed to preside over the alimentary secretion. To the better understanding of this subject, it is necessary to remember that each and every portion of the body is connected with the brain and spinal axis by two different sets of nerves, *viz.*, the afferent and the efferent.* The afferent set of nerves send impressions from all parts of the body, through the ganglionic and spinal centres, up to the brain or sensorium; while the efferent set send impressions out from the brain, through the same spinal and ganglionic systems, to all parts of the body. Indeed, it is confidently asserted by physiologists, that each and every part of the body must have its peculiar centre in the brain, and its peculiar nervous fibril to maintain a connection with that centre, though a number of these fibrils may be included within one general sheath. If any irritation, therefore, occur in any portion of the body, as, for example, in the alimentary canal, the afferent nerves, which are extended, in a delicate plexiform expansion, all over the intestinal mucous surfaces, immediately take cognizance of the fact and instantly convey the intelligence, in the form of a sort of telegram, up to the brain, or capitol; while the brain, thus informed, loses no time in sending down, through the efferent set of nerves, such reinforcement is as necessary, or the best at command, to relieve the difficulty. Thus, it must be borne in mind, that all parts of the body are bound *intimately* together by numerous bonds of sympathetic union, so that all parts are under the constant vigilance of sleepless sentinels, ever ready to send up to the brain any information, and warn it of any danger; while the sensorium, like a watchful governor, is constantly on the *qui vive*, with a numerous retinue of prompt and willing servants, in the shape of the efferent nerves, ready to execute the will and remove from, or ward off, any danger to life or limb. No person can fully understand the diseases to which I am now directing attention, until he has carefully studied that complicated system

* The discovery of these two sets of nerves, is due to Sir C. Bell, in 1835.

of reflex action so ably elucidated by Marshal Hall, and extended and amplified by Dr. Frazer Campbell and M. Claud Bernard. [See Note.]

It is the excito-secretory system of nerves which, I think, plays so conspicuous a part in the diseases above mentioned. When the nerve centres presiding over nutrition, digestion, and the intestinal secretions generally, from any cause (and they are subject to be sympathetically modified in their molecular action by the slightest and most varied impressions upon any portion of the body), become too much depressed, irritated, or *in any way* so modified in the delicacy of their normal structure as to unfit them for furnishing the stated necessary nervous supply, *disordered digestion is the result*—either diarrhœa or constipation. Perhaps they may succeed and alternate with each other. When *constipation* is the result, especially if obstinate and long continued, nature attempts, by a well-known law in the conservation of the animal economy, to do *indirectly* what she has failed to do *directly*. She attempts to relieve the constipation or loaded state of the bowels, by promoting the absorption of the fæcal matters and carrying them off through the skin and kidneys. In doing this at such a disadvantage, and in such an unnatural way, it is not surprising, sometimes, to see the whole system poisoned, the fluids corrupted, and the *nerve centres* still further depressed or irritated, and more especially when it is remembered that these nerve centres *first* feel the noxious effects of any toxic agent in the circulation, that they, more than any other tissue in the body, demand, for their normal action, pure, oxygenated blood, free from any deleterious agents whatever, we shall be able the better to understand why they are so frequently at fault. When the importance of these delicate structures is better understood in maintaining the *solidarity* of the whole human economy—the muscular tension throughout the body, the part they play in the processes of elaboration, secretion, growth, repair, and extension; disintegration, excretion, respiration, digestion, locomotion, and ratiocination, we may begin to conceive something of the magnitude and importance of this intricate subject.

Now, such a patient, if not relieved by some *coup de main*, some change in habits, regimen, climate, or medicine, *sinks*, inevitably, into a state of cachexia, or some form of disease justly alarming to himself and his friends. Now, I wish to call the attention of medical men here to this matter, with the view of obtaining their assistance in tracing the chain of morbid action, link by link, up to the prime cause and true pathology of many of these anomalous cases.

When it is borne in mind that, in this climate especially, the onus of disease is thrown upon the nerve centres, and that it is in these nerve centres that the nerve force is correllated into cell action, secretion; *here*, that the nerve force seems to be generated, in fact, and that the nerve centres, *altogether*, preside over all the physical phenomena in the body, it will not seem so strange that even slight molecular changes in these delicate structures should crop out in grave and formidable symptoms of disease, nor, moreover, in the different stages of the same disease that there should often be very different signs displayed to the mere superficial observer. For instance, in the first stage of a case of constipation, caused by a depressed state of the sympathetic ganglia, so that the intestinal mucous surfaces supplied by the nervous expansions from these nerve centres, feeling the want of the normal nervous *supply*, sends up, through the afferent nerves, to these nerve centres a demand for the necessary power to carry on their functions, while the mucous surfaces, from a want of the proper nervous distribution, by a well-known law in pathology, are thrown into such an abnormal state of excitement as to *intensify* this *demand*, and thereby call out, temporarily, the *reserved forces*, in such a way as to induce an extra effort to relieve the loaded bowels of their unwonted load. Perhaps they do partially succeed, but when this urgent demand is relieved, they sink back into a still greater degree of torpor and depression than before. The bowels fill up again very soon, when nature, no longer able to relieve the distension in the natural way, resorts to another and less direct method of relief, *viz.*, the absorbents at once take up all matters of sufficient tenuity to be absorbable, while the more crude and irritating contents remain hardened and impacted in the folds of the colon till nature, arousing herself again, makes another desperate effort to get rid of the offending mass in the natural and direct way. She may even again partially succeed, and thereby squeeze through between the scybalous masses the more fluid contents of the alimentary canal, inducing the belief in the mind of the unwary physician, perhaps, that the patient has a diarrhœa, and he resorts to such medicines as effectually put a stop to even these feeble efforts of *nature.*

Now, if the real cause of the difficulty could be divined, and the nerve centres, in these cases, could be steadily aroused and permanently sustained in a more exalted action, the good results of the means employed would soon appear. But now, if nothing is done in the right direction that reaches the true cause of the difficulty, as soon as these excrementitious and noxious matters absorbed, enter the circulation they exert a deleterious and poisonous influence upon the whole system, and the nerve centres, in common with the whole organism, suffer still further injury and depression. Now, if the patient happens to be a pregnant female (which often occurs with ladies in such a condition), she goes on halting, complaining, and suffering from the effects of these sources of blood-poisoning and secretion-corrupting, until her vital powers of resistance are so diminished that, when the critical changes attending and consequent on confinement take place, she finds herself illy prepared to go safely through the trying ordeal. Now, mark what happens to her. She sinks into that dangerous form of puerperal fever denominated *putrid*, where all the symptoms of a zymotic poison, so much dreaded, occur. Every person who has practised midwifery any considerable time, must have been occasionally called to witness such cases of malignant putrid puerperal fever, where the powers of vital action were so impaired from some previous habit of body, as to be able to make no effectual stand against the terrible destroyer.

In these cases, there is often great doubt and serious misgivings in the mind of the medical attendant, in regard to the nature of the disease and the best mode of treatment. There is often undeserved censure, also, cast upon the physician, from the real cause of the trouble not being understood. My experience induces me to believe that constipation *before confinement* is a frequent and strongly predisposing cause of this dangerous disease. Several cases have come under my observation, where I happened to know that such a state of the bowels preceded the confinement. In running my mind back over a professional life of some twenty years, I cannot now recal a single case of such severe and dangerous fever occurring, where the bowels had been properly attended to before labor. I do not pretend to say that this is the *sole* cause, for I am satisfied that it is not, but I do firmly believe that if women were more careful in this particular during the latter months of pregnancy, far less cases would occur, and those that do happen would be milder and more manageable than they are. Should I be called to a case of the kind I am speaking of, and, on enquiry, find that the bowels had been neglected before confinement so that there had been serious habitual constipation, I declare to you, gentlemen, that this fact *alone* would have much influence in the prognosis. I am the more anxious to impress this point on the minds of the profession because, as I have intimated already, I have noticed a disposition among some of my co-laborers in the practice of medicine to ignore purgatives in the treatment of all diseases. From so limited a means of observation, I acknowledge that it would not be safe for me to be too sweeping in my conclusions; nevertheless, I am quite confident, from what I have seen, that there are numerous cases of delicately organized females, and even males, of sedentary habits, among the young, and more especially among the *older*, who are groaning, unrelieved, under a complication of diseases of a somewhat anomalous character, who would be quickly restored to health and happiness by simple means, if the fact could only be clearly seen by the physician, that it is *altogether impossible* to *arouse* these *nerve centres* until the *influx* of *putrescent matters* from the *alimentary canal* are stopped and turned off in their *natural channel.* One of the first, most practical, and most impressive lessons I learned in the study of female diseases was from Dr. Gooch, in regard to dysmenorrhœa and some forms of menorrhagia attended with pain, where he says that the best and most effectual way to arrest the discharge often is to *stop the pain* and let the discharge take care of itself. By following this advice and directing my remedies to the relief of the pain, by acting on the *uterine ganglia* and nervous system, I have sometimes cured distressing cases with great ease.

Several years ago, I was called to see a young man whose bowels could not be got to move, although he had taken a large amount of active cathartics and injections. This obstinate constipation occurred in consequence of a kick from a horse in the abdomen. After all other means seemed to fail, and while I was waiting for a consultation, it occurred to me that the difficulty might arise from a sort of paralysis from concussion of the sympathetic ganglia, and I was led to this conclusion from the fact that a blow upon the epigastrium would produce syncope; I therefore determined to try the battery. In the meantime Dr. Worthington came, who, regarding it as a case of obstruction from local inflammation, advised bleeding and a blister. But while we were parleying over the matter, the father came out and told us the boy was all right, as his bowels had been thoroughly moved. This changed the phase of the case, and he got well without bleeding or blistering. Did anybody ever before hear of concussion of the sympathetic ganglia? And why not?

NOTE.—Dr. Marshal Hall, of London, published his discovery of the excito-motory system of nerves in 1837. In 1850, Dr. Henry Fraser Campbell, of Augusta, Ga., announced the discovery of a similar relation between the cerebro-spinal, and the great sympathetic, or that extensive system presiding over the functions of nutrition, elaboration, and secretion, and he called it the excito-secretory system of nerves. In 1855, M. Claude Bernard, of Paris, made his interesting experiments upon the pneumo-gastric nerve, and showed that it influenced, very much, the secretion of the liver, and tended, therefore, to confirm, by actual experiment, the previous discovery of Dr.

Campbell. In 1857, Dr. Marshal Hall, after acknowledging the honor due to these two men, announced his belief that, in addition to his original discovery of the primary system of reflex action, called excito-motory, there may be several sub-systems, and he thinks the excito-secretory should be classed with the latter. Now, while I acknowledge that the excito-motory system may be so extended as to comprehend all the offices and function of the body, as every act, every molecular change even, implies motion; and every excitor influence, mediate, immediate, or remote, voluntary or automatic, internuncial, or imperative, must take place through the nervous system; yet, I see no advantage in thus extending it; and I think it more natural to make divisions and subdivisions according to functions and offices, and to generalize, also, accordingly. Whether we multiply the nerve centres and divide the spinal axis into separate segments, when the circle is completed through them, or take a more general view of the whole organism, classing together corresponding offices and functions, is very much a matter of taste where the *modus operandi* is the same, and where they have alike for their object the conservation of the animal fabric.

Therefore, in order to facilitate the study, and systematize the matter more thoroughly, I propose to make several primary systems and several sub-systems. I would confine the primary excito-motory system to the muscular movements of the body, and make the sub-systems include the voluntary and involuntary. The primary excito-secretory system should include all impressions and causes, transmitted through the nervous system, influencing, in any way, the various secretions of the body. The sub-systems under this head may be extended almost indefinitely. Other primary systems may be indicated, as the excito-circulatory, general and local: while we may have still others, as the excito-inflammatory, excito-suppuratory, etc. If it be objected that there are, in fact, no such actual systems in the anatomy of the human body, I will remark that if it be true, as asserted, that every portion of the body has its distinct centre in the brain or spinal axis, and a distinct filament to maintain that connection; when we see certain impressions upon *one* part of the body produce, *uniformly*, the same effects upon some *remote organ or tissue*, we have good reason to believe that there subsists, between these two parts, a connection closer and more intimate than with other portions of the body. This connection must be maintained, somehow, through the nervous system. Common observation in the effects of remedies, and in physiology and pathology, all concur in establishing this truth, and, to the physician, a thorough knowledge of these intimate relations and remote sympathies is important and doubly interesting. In order, therefore, to bring this matter strongly and more fully before the mind, so that a bird's eye view can be had, I propose to arrange the subject in a table, as follows:

| | | |
|---|---|---|
| Excito-Ideational | | All impressions received through the sensorium on the cerebrum, exciting or modifying the thoughts. |
| Ex-Motory | Voluntary | Ideo-motor and emotional influences, having the the cerebrum for their centre. All the influence the will may exert in the movements of the body. |
| | Involuntary | Sub-Systems— |
| | | Ex-Respiratory, as the atmosphere, or cold water on the body. |
| | | Ex-Emetory, Emetics, sights of disgusting objects. |
| | | Ex-Deglutory, Food masticated and insalivated. |
| | | Ex-Defecatory, Fæcal matter in the rectum. |
| | | Ex-Parturient, Full period of utero-gestation. |
| | | Ex-Convulsive, Worms, aura epileptica, remote irritations. |
| Ex-Secretory | | Ex-Salivary, Food in the mouth. |
| | | Ex-Gastric, Food in the stomach. |
| | | Ex-Biliary, Calomel, the passage of the alimentary mass. |
| | | Ex-Lactary, Empty uterus, Faridisation, etc. |
| | Ex-Follicular | Dermo-perspiratory and sebaceous-mucous. All the gl'ds whose ducts open into the al'y can'l. |
| Ex-Circulatory | General, as | Stimulants, healthy food, pure air, mental habitudes. |
| | Local | Hyperæmia, local determinations, congestions, etc., in health or disease, from the blush on the cheek to the gravest forms of disesase. |
| | | Metastasis, retrocessions, remote and secondary inflammations, as pneumonia from burns, etc. |
| Ex-Suppuratory and Ex-Inflammat'y | | It is often observed that remote irritations produce inflammations. Sometimes this inflammation runs rapidly into suppuration, as in case of metastatic abscess, while in other cases, as in orchitis from mumps, this result rarely happens. |

Why is this? There must be some occult cause for this, and I know no better explanation, than to say that the nervous distribution is such, that in one case there is a tendency to suppuration, and in the other, though the inflammation may be equally new, still as the suppurative tendency being wanting, resolution finally takes place. Whether this explanation is correct or not, I think this table will assist to fix the fact on the mind of the *pupil* or physician, and assist him to investigate the matter, until he obtains a better one.

### ESSAY.

"VIA OFFICII VIA SALUTIS."

After my appointment as delegate to attend this Society, I thought it might be expected of me to contribute something to promote the interest of the convention; and I felt, also, a desire to cast in my modicum of influence in some way, to advance the general good. Therefore, after a little deliberation, I determined to present, for the consideration of this meeting, the above motto, which, translated, means, "The path of duty is always the path of safety." In the practical illustration of this motto, allow me, gentlemen, to present to you this morbid specimen, together with the following brief history of the case:

Mrs. G. R. B., relict of the late Dr. B., of Schuyler County, Ill., *æt.* 31, the mother of three children, and a widow for some 18 months, had been suffering for about a year from repeated and exhausting attacks of uterine hemorrhage, for which she had been subjected to various modes of treatment by the physicians of the various schools, and at different places. First, at Quincy, Ill., and, subsequently, at Rushville, Schuyler County, Ill., where she took no small amount of cayenne pepper, lobelia, etc., according to the most approved system of modern eclecticism; and, I believe, "infinitessimals" *ad infinitum*, from the disciples of Hahnemann's theory of "*similia similibus curantur*," till she was reduced to the very verge of the grave.

The disease had been pronounced, either through ignorance, or by way of justification for the inefficiency of their treatment, by these modern medical wiseacres, "cancer of the womb;" and she had, consequently, taken the thousand and one infallible cures and specifics for that hopeless affection, till at length, disheartened in mind and exhausted in body, pallid and ensanguined, thoroughly discouraged, and quite disgusted with the various unavailing modes of treatment brought into requisition for her relief, she was, at length, brought home to her father's house to meet her fate, despairing of all cure; when, on the 19th of November, 1864, I was first called to see her.

On examination per vaginam, I felt what I then took to be the posterior lip of the os uteri, somewhat swollen and elongated, but soft and *insensible to the touch*, and the vagina filled with coagula. She had a rapid, thread-like, and hemorrhagic pulse, frequent and troublesome palpitations, with that peculiar feeling of vertigo so characteristic of excessive loss of blood. Not feeling quite satisfied about the diagnosis, after several days of delay, however, a consultation was advised. Dr. E. C. Parker, an intelligent physician of Beardstown, Ill., was called to meet me at her bedside. After a review of the symptoms, he gave it as his opinion, that the hemorrhage proceeded from the internal surface of the uterus; and he advised an injection of diluted creosote. In making the injection I used the speculum, for the double purpose of convenience in the operation and the more perfect examination of the os and neck of the uterus. The first sitting satisfied me that what I had taken for the posterior lip of the uterus, enlarged, was, in reality, a tumor of some kind, about as large as a *filbert*, projecting through the os. It was of a pinkish color, and seemed to be covered with a villous coating. It was insensible to pressure, and, indeed, it had never caused her any pain in that region. I pressed the canula up through the os, in front of the tumor, into the uterus, and completed the injection; but it had no effect whatever on the hemorrhage. Hence, at another sitting, a few days afterwards, I painted the tumor all over with concentrated creosote, causing no pain worth mentioning, which arrested the flooding for the space of two weeks. At the expiration of this time, it returned as before. On examination, I then found that the tumor had increased nearly one-half in size during that short time. I now felt fully satisfied that the hemorrhage proceeded from the tumor, in fact, oozed out from all parts of its surface. From this time, my mind was made up that it was a polypus, and I strongly suspected, *believed*, indeed, that the suppression of the discharge had contributed very much to the growth of the tumor.

Now, gentlemen, I cannot do justice to myself in this statement, without entering a little more *in extenso* into the circumstances connected with this case, by way of explanation. The family with whom this lady stood connected lived near me, and had been, for *more than ten years*, my most persistent and malignant professional enemies; and they had never, during all that time, lost any opportunity of traducing my professional character, or prejudicing all they could possibly influence (and they exerted no small influence, I assure you) against me, and especially our system of practice. The *rationale* may be found in the fact, that the husband of this lady had been, when alive, an eclectic physician, practising in the same part of the county. After his death, my relations with the family became more amicable, though my bearing towards them remained quite the same (always courteous and affable), so that they had several times requested me to visit members of the family in cases of sickness. Add to this the additional fact that I had sold out my property and practice in that neighborhood, and intended soon to leave the county. I felt, therefore, no further desire of extending my reputation as a physician in that vicinity. I supposed, also, that she would be very likely to plead the protection of the profession; and last, though not least, I knew that to perform the operation of removal by ligature, which I considered the most expedient under the circumstances, I should be under the necessity of sending off some distance to procure the instruments I deemed necessary, *viz.*, a speculum, matrices, and Gooch's double canula, as they were not owned by any physician within my reach. Notwithstanding all these motives prompting me to *cut my patient* (and I knew that these circumstances would make my excuse plausible) and leave her to her fate, or to the very great inconvenience of seeking medical aid elsewhere, which, I fully understood, would be exceedingly difficult, *I felt prompted by every principle of honor and fidelity, in her great need and extremity, to remember nothing of the past,*

but to make whatever sacrifice of *feeling, time,* and *money* was necessary, and go forward and render her the necessary assistance, with the hope of saving the life of a mother and a daughter, and thereby make a *generous* and *magnanimous* return for all the sharp and unkind cuts of the past.

After a little deliberation, I determined to make "a clean breast" of the matter, and I, therefore, told her and her friends, in full household, the precise nature of the disease, what I proposed to do, and what would be the *probable result.* Let it be remembered, that I had not before given a definite opinion in the case, and they had been led to believe the disease incurable or malignant; hence, they were all very agreeably surprised to learn that the case was capable of being cured, and their *faces brightened up* and became *radiant with hope and joy,* as I represented to them the probability of her *restoration* to *health,* to *happiness,* and to *them.* I stated, also, to them, that if they doubted my opinion, or the feasibility or propriety of the operation, they might call additional advice from any source they chose. After a little deliberation and a short conference, they seemed cheerfully to resign the case into my hands, and even encouraged me to go forward and do what I thought was necessary and right in the matter, and they would be satisfied with the result. I, therefore, sent immediately to Chicago and procured the aforesaid instruments, and, on the 10th of March, 1865, I ligated the tumor at the neck, which came off on the fifth day, without an untoward symptom; since which time she has fully regained her health, and continues to enjoy the same up to this time.

Thus, I have my *revenge* in the *double pleasure* it now *affords me,* of being able to present to you, gentlemen, *this beautiful specimen of uterine polypus,* and moreover, of giving to you an instance of the practical exemplification of the truth of the motto I have selected as the subject of these remarks, which, perhaps, we are too prone to forget, *viz.,* that the path of duty and the path of honor are, always, paths of safety and success.

Perhaps I should not omit to state, in connection with the history of this case, that this lady always enjoyed good health up to the time of the death of her husband. During her maiden and married life no irregularity amounting to disease had ever occurred in her uterine functions; but immediately on the appearance of the hemorrhage the menses became suppressed, and very soon thereafter, her appetite became depraved and capricious, with a constant longing after, and an irresistible desire for, slate pencils, soapstones, etc.; but, on the removal of the tumor, the menses very soon returned, and the anæmiated and chlorotic countenance gave place to the ruddy hue of health and vigor, and all that vitiated, voracious appetite vanished as by magic.

If this brief and imperfect sketch shall, in any degree, serve to encourage the members of our noble profession who might otherwise be tempted to forget their dignity, in seeking redress by retaliation for personal wrongs and professional injustice, to *bide their time*, turning neither to the *right* nor the *left*, but to pursue, *straight forward* in the path of duty and the path of honor, the *even tenor of their way*, with the *full assurance that true honor*, under all circumstances in life, *will*, sooner or *later*, *inevitably bring its legitimate and ample reward*, I shall have written these lines at the right time, and read them in the right place. I close, as I began, with the motto, "*Via officii via salutis.*"

